# Discovery, activity and characterisation of an AA10 lytic polysaccharide oxygenase from the shipworm symbiont *Teredinibacter turnerae*

**DOI:** 10.1186/s13068-019-1573-x

**Published:** 2019-09-30

**Authors:** Claire. A. Fowler, Federico Sabbadin, Luisa Ciano, Glyn R. Hemsworth, Luisa Elias, Neil Bruce, Simon McQueen-Mason, Gideon J. Davies, Paul H. Walton

**Affiliations:** 1 Department of Chemistry, University of York, York, YO10 5DD UK; 2 Department of Biology, Centre for Novel Agricultural Products, University of York, York, YO10 5DD UK; 3 Present Address: School of Chemistry and Photon Science Institute, University of Manchester, Oxford Road, Manchester, M13 9PL UK; 4 Present Address: Astbury Centre for Structural Molecular Biology and School of Molecular and Cellular Biology, Faculty of Biological Sciences, University of Leeds, Leeds, LS2 9JT UK

**Keywords:** Oxygenase, Enzyme, Cellulose, PMO, LPMO, Shipworm, 3D structure, CAZyme

## Abstract

**Background:**

The quest for novel enzymes for cellulosic biomass-degradation has recently been focussed on lytic polysaccharide monooxygenases (LPMOs/PMOs), Cu-containing proteins that catalyse the oxidative degradation of otherwise recalcitrant polysaccharides using O_2_ or H_2_O_2_ as a co-substrate.

**Results:**

Although classical saprotrophic fungi and bacteria have been a rich source of lytic polysaccharide monooxygenases (LPMOs), we were interested to see if LPMOs from less evident bio-environments could be discovered and assessed for their cellulolytic activity in a biofuel context. In this regard, the marine shipworm *Lyrodus pedicellatus* represents an interesting source of new enzymes, since it must digest wood particles ingested during its natural tunnel boring behaviour and plays host to a symbiotic bacterium, *Teredinibacter turnerae*, the genome of which has revealed a multitude of enzymes dedicated to biomass deconstruction. Here, we show that *T. turnerae* encodes a cellulose-active AA10 LPMO. The 3D structure, at 1.4 Å resolution, along with its EPR spectrum is distinct from other AA10 polysaccharide monooxygenases insofar as it displays a “histidine-brace” catalytic apparatus with changes to the surrounding coordination sphere of the copper. Furthermore, *Tt*AA10A possesses a second, surface accessible, Cu site 14 Å from the classical catalytic centre. Activity measurements show that the LPMO oxidises cellulose and thereby significantly augments the rate of degradation of cellulosic biomass by classical glycoside hydrolases.

**Conclusion:**

Shipworms are wood-boring marine molluscs that can live on a diet of lignocellulose. Bacterial symbionts of shipworms provide many of the enzymes needed for wood digestion. The shipworm symbiont *T. turnerae* produces one of the few LPMOs yet described from the marine environment, notably adding to the capability of shipworms to digest recalcitrant polysaccharides.

## Background

Harnessing the activity of enzymes for the breakdown of cellulose and related plant-cell wall polysaccharides is important in the quest for environmentally sustainable fuels in the form of second-generation biofuel, from cellulosic wastes and bespoke energy crops. In this context, one of the most significant breakthroughs has been the discovery [1–3] of chain-breaking “lytic” polysaccharide monooxygenases (LPMOs, sometimes PMOs) that oxidatively deconstruct recalcitrant polysaccharides, most notably cellulose, chitin, hemicelluloses and retrograded starch (generic LPMOs have been extensively reviewed, examples include [4–8]).

When used as part of enzyme cocktails, LPMOs significantly boost the activity of classical glycoside hydrolases, thereby offering great potential for the sustainable breakdown of recalcitrant biomass within a commercial setting. As such, there is an on-going search for new variants of LPMOs, particularly from organisms which are effective and voracious decomposers of biomass (for example [9]).

Until now, most cellulose-active LPMOs have been isolated and characterised from wood-decaying fungi and soil bacteria. As a complement to these sources of new enzymes, more complex animals and their microbial symbionts present a different biological context for biomass-enzyme discovery. In the present work, we examined marine xylophagous bivalve molluscs—called shipworms—that burrow through submerged wood from which the organism derives nutrients, mainly sugars [10, 11]. Shipworms are a major cause of damage to submerged timber structures.

Within their gills, shipworms harbour communities of endosymbiotic bacteria in specialised eukaryotic cells (bacteriocytes) [12]. Recent transcriptomics and proteomics analyses of dissected organs revealed that wood digestion in shipworms is accomplished through the combined action of enzymes of both endogenous and symbiotic origin [13, 14]. These studies demonstrated that shipworm gill endosymbionts produce a multitude of carbohydrate active enzymes, in which the bacterial genes coding for predicted (CAZY family, http://www.cazy.org [15]) AA10 LPMOs and GH6 cellobiohydrolases are among the most highly expressed. Compositional analysis of undigested wood and shipworm faeces (frass) also revealed that, while hemicellulose and lignin were virtually unaffected, over 40% of the cellulose was removed after digestion by the shipworm *Lyrodus pedicellatus* [13]. Such high levels of activity signal that the constituent enzymes may be particularly fruitful targets within the context of the on-going search for biomass degrading enzymes for biofuel production.

Here, we report the in-depth biochemical and structural characterisation of the first LPMO from a shipworm endosymbiont (*Teredinibacter turnerae* [16]), the recombinant protein of which was produced heterologously in *Escherichia coli*. Through mass spectrometry and HPLC analysis of released products, we show that this enzyme has mixed C1–C4 oxidising activity on cellulose and boosts the breakdown of this recalcitrant polysaccharide by glycoside hydrolases. X-ray diffraction and multi-frequency EPR spectroscopy studies reveal a near classical AA10 LPMO secondary/tertiary structure, yet one that contains a second Cu-binding site. The coordinating residues of both copper sites are conserved in close homologs of *Teredinibacter turnerae*. The histidine brace site, composed of the N-terminal histidine and a second histidine side chain, is conserved across nearly all known LPMOs. The second Cu site is, however, not conserved across the wider LPMO family and its function within the *Teredinibacter turnerae* LPMO is unclear, although it may be related to charge transfer pathways through the protein.

## Results

### Expression and enzymatic characterization of the AA10 LPMO from *T. turnerae*

**Fig. 1:**
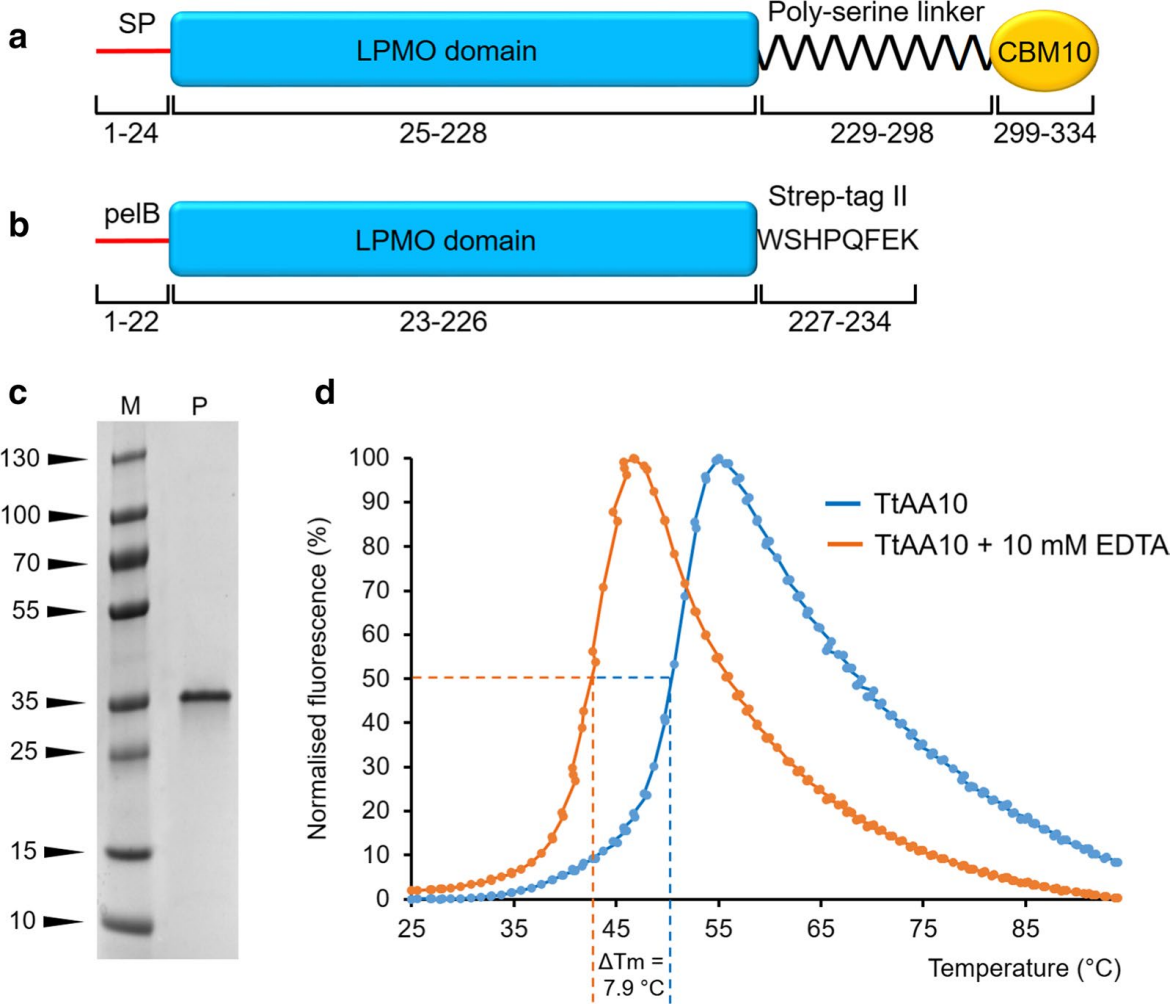
Production and stability of *Tt*AA10A. **a** Architecture of the full-length *Tt*AA10A protein, featuring a signal peptide for secretion (SP), an AA10 LPMO domain, a 70 residue poly-serine linker (predicted to be flexible) and a predicted CBM10. **b** Architecture of the recombinant *Tt*AA10A core used in this study. **c** SDS-PAGE of purified *Tt*AA10A (LPMO domain) heterologously produced in *E. coli* (*M* molecular weight markers in kDa, *P* purified protein). **d** Thermal shift analysis of purified *Tt*AA10A LPMO domain, showing the destabilising effect of copper removal through EDTA treatment, causing a 7.9 °C decrease of the melting temperature

The gamma-proteobacterium *T. turnerae* is the only endosymbiont found within the shipworm gills to have been successfully isolated, cultured and had its genome mapped [16]. Through automated annotation and manual BLAST [17] searches of the predicted *T. turnerae* proteome, we identified one gene (NCBI Reference Sequence: WP_019602454.1) coding for an AA10 LPMO (hereafter *Tt*AA10A). The predicted protein sequence features an N-terminal signal peptide, the LPMO domain and a serine-rich linker region followed by a carbohydrate binding module (CBM) 10 domain (Fig. 1a). AA10s have been found with appended CBM2, CBM3, CBM5, CBM10, CBM12, CBM18 and CBM73 domains (Bernard Henrissat personal communication) and are known to be active on cellulose or chitin. CBM10 domains are thought to be cellulose binding and so may provide cellulose recognition that is unlikely to be associated with a catalytic event [18]. Whilst different from the CBMs commonly found attached to AA10 proteins [19], its presence in the domain structure of the *Tt*AA10A gene gives an indication that this protein may be primarily active on glucose-based polysaccharides.

After multiple attempts to express the gene with various affinity, solubility tags and different secretion signals, sufficient protein for analysis was finally obtained through the production of a C-terminally strep-tagged LPMO catalytic domain (from His25 to Gly228) in *E. coli* (Fig. 1b). The purified tagged protein was loaded with excess copper, de-salted through size-exclusion chromatography, analysed for purity through SDS-PAGE (Fig. 1c) and mass spectrometry-based protein ID (not shown), and used for subsequent experiments.

Recombinant *Tt*AA10A (catalytic domains, 25–228, only) displays the hallmarks of a correctly folded AA10. Thermal shift analysis (Thermofluor) of purified, Cu-loaded *Tt*AA10A indicates a melting temperature (*T*_m_) of 50.4 °C. Stripping copper with 10 mM EDTA lowers the *T*_m_ to 42.5 °C, suggesting a protein stabilising effect by the metal cofactor, as reported in previous literature for other LPMOs (for example [20, 21], Fig. 1d). We also noticed a variability in protein preparations, with some preparations containing a single (active-centre) Cu, whilst others contained two Cu atoms, described below.

**Fig. 2:**
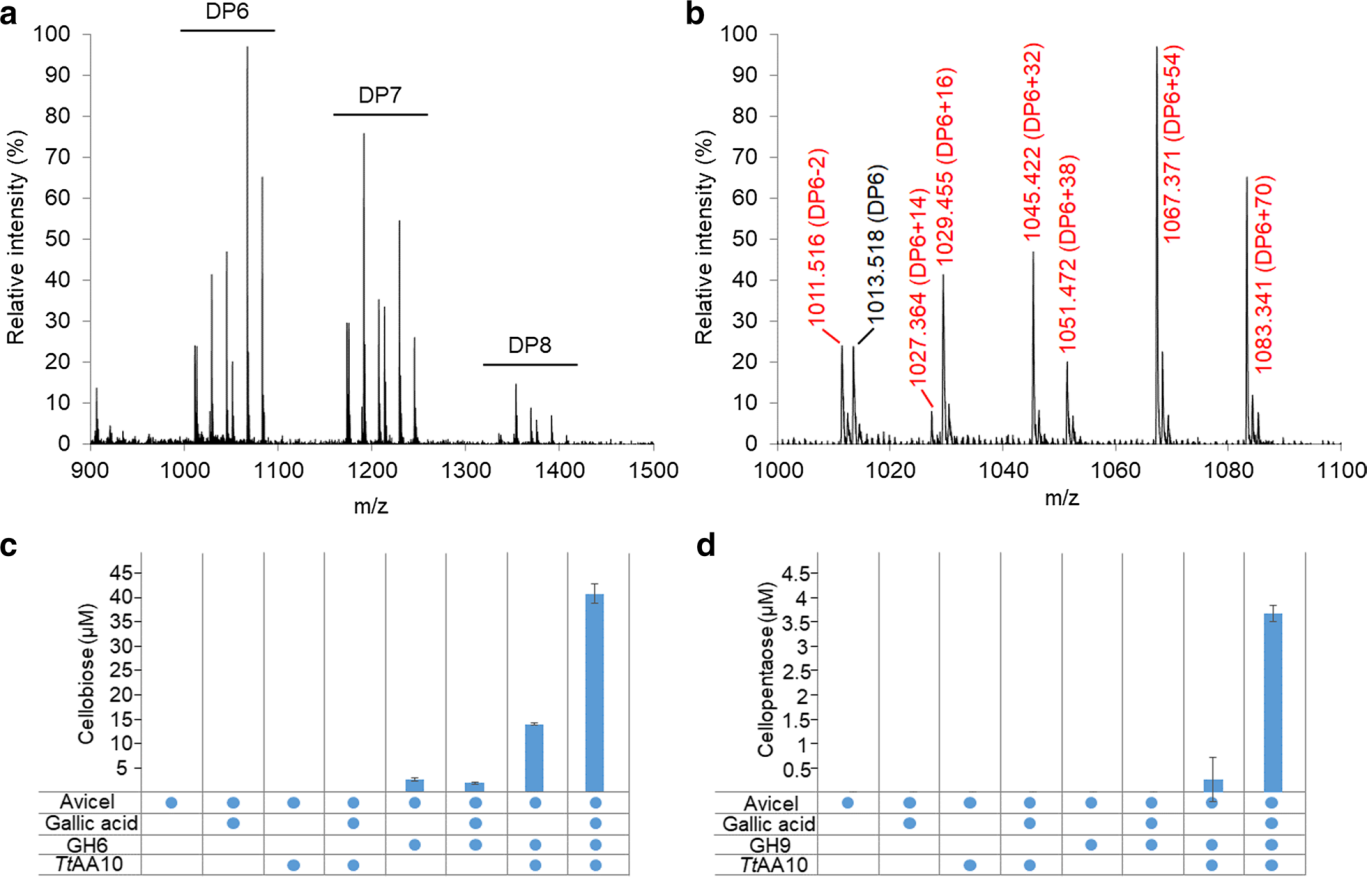
Activity of *Tt*AA10A on polysaccharides. **a** MALDI-TOF MS spectrum of products obtained after incubation of 4 mg/mL Avicel with 2 µM LPMO and 4 mM gallic acid, showing native and oxidised oligosaccharides. The main peaks correspond to: C1 or C4 keto adduct, monosodiated adduct (− 2 species); C4 keto plus C1 aldonic acid, monosodiated adduct (+ 14 species); C1 aldonic acid or C4 gemdiol, monosodiated adduct (+ 16 species); C4 gemdiol plus C1 aldonic acid, monosodiated adduct (+ 32 species) and disodium adduct (+ 54); C1 aldonic acid, disodium adduct (+ 38 species). An additional peak with mass 1083 *m/z* could not be reliably assigned to any known product of LPMO oxidation and was tentatively interpreted as a higher oxidation level at the C6 (+ 70 species, corresponding to C4 gemdiol plus C1 aldonic acid plus C6 aldonic acid, disodium adduct). Native and oxidised species are marked in black and red, respectively. Relative intensity represents 1.23 × 10^3^. **b** Expanded mass spectra for DP6. Synergy experiment showing the release of cellobiose from microcrystalline cellulose (Avicel) by a commercial GH6 (**c**) and of cellopentaose by a commercial GH9 (**d**). The LMPO significantly boosts the activity of both glycoside hydrolases, and such effect is increased by addition of gallic acid

Activity assays, on both single and double Cu site samples, were carried out on a range of commercial polysaccharide substrates (Avicel, β-chitin from squid pen, α-chitin from shrimp shell, cellohexaose, corn starch, pachyman, beechwood xylan, glucomannan, xyloglucan, lichenan, galactan, galactomannan and mannan) in the presence of the reducing co-factor, gallic acid. Samples were analysed after 24 h by MALDI-TOF MS and peak masses of the reaction products compared to previously published data, revealing a mixed C1–C4 oxidation pattern, exclusively on cellulose, and dependent on the presence of the electron donor (Fig. 2a, b). Products were not detected in any of the negative controls (Additional file 1: Figure S1). MALDI-TOF MS analysis of crude extract from activity assays carried out with Cu-loaded *Tt*AA10A in the presence of 10 mM EDTA failed to detect the release of products (data not shown), indicating that, as expected, copper is essential for activity.

Synergy experiments were performed by co-incubating *Tt*AA10A and commercial glycoside hydrolases (GH6 and GH9) in the presence of Avicel and gallic acid, and the resulting mono- and oligosaccharides were quantified using high-performance anion-exchange chromatography (HPAEC). While reactions containing either LPMO or GH alone released negligible amounts of free sugars, co-incubation reactions showed a strong synergistic effect, further enhanced by the presence of the electron donor (Fig. 2c, d, Additional file 2: Figure S2). It is worth noting that both commercial GHs (GH6 and GH9) tested during these experiments belong to families that were identified as among the most abundant in the digestive proteome of shipworms [13], strengthening the biological relevance of the abovementioned activity assays in the context of wood digestion in the shipworm milieu.

### Electron paramagnetic resonance spectroscopy

**Fig. 3:**
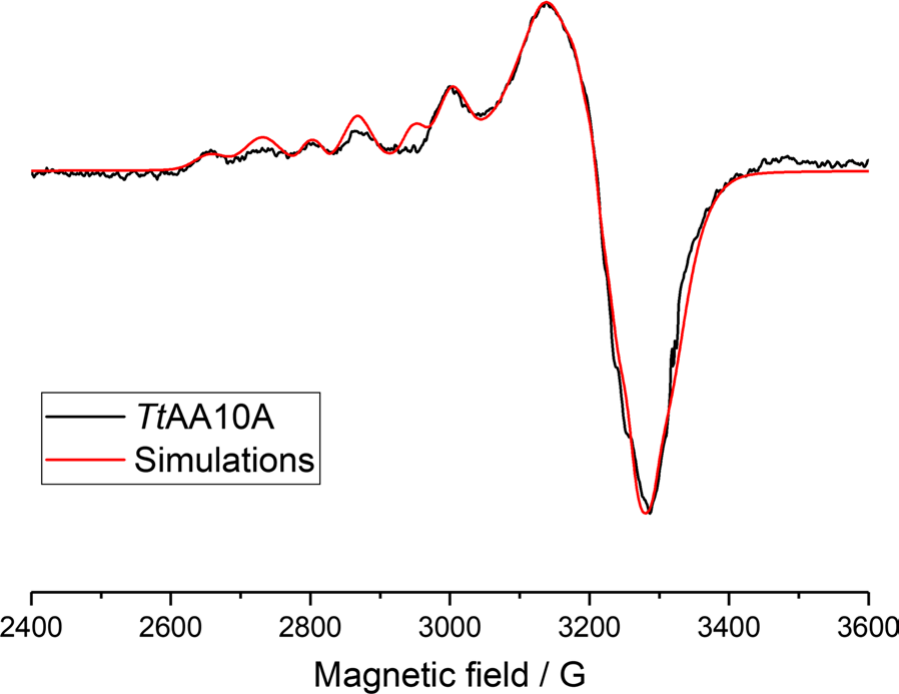
CW X-band EPR spectrum of copper-saturated *Tt*AA10A. Simulations obtained using the parameters reported in Table 2 for Species 1 and the following values for Species 2: *g*_*x*_ = 2.03, *g*_*y*_ = 2.07, *g*_*z*_ = 2.314, |*A*_*x*_| = 40 MHz, |*A*_*y*_| = 60 MHz and |*A*_*z*_| = 465 MHz with addition of one coupled N atom with *A*_N_ principal value of 35 MHz

Our first evidence that some protein preparations contained two Cu sites came from EPR analyses. The frozen solution (165 K) X-band CW-EPR spectrum of Cu-saturated *Tt*AA10A (Fig. 3) exhibited two sets of hyperfine peaks in the parallel region of the spectrum, indicating the presence of two distinct copper coordination geometries, arising either from different coordination environments within a single site (e.g. differences in protonation states of ligands) or a distinct second copper-binding site. Indeed, an accurate simulation of the parallel region of the spectrum could be obtained with two different species, each of which afforded a different set of spin Hamiltonian parameters, *g*_*z*_ = 2.267 and |*A*_*z*_| = 425 MHz (species 1), and *g*_*z*_ = 2.314 and |*A*_*z*_| = 465 MHz (species 2), Table 1, with a ratio between species 1 and 2 of approximately 3:2. The *g*_*z*_ value of species 2 is high compared to what one might expect for the typical AA10 LPMO copper coordination in the active site (spectroscopy of LPMOs recently reviewed in Ref. [20]), on the basis of which we assign species 1 to a copper bound to the canonical histidine brace active site. Its spin Hamiltonian values are typical of an axial Cu coordination geometry which contains a mixture of N and O-donating ligands [22]. (Note that species 2 cannot be from a free copper species in solution as all small molecule species are removed during the protein preparation; hence, all copper signals in the EPR arise from protein-bound copper.)

**Table 1:** Spin Hamiltonian parameters (parallel region) of species 1 and species 2 from sample shown in Fig. 3

	Species 1	Species 2
*g*_*z*_	2.267	2.314
|*A*_*z*_| (MHz)	425	465

To determine whether the two signals arose from a single copper-binding site with different coordination geometries, or from two distinct copper sites, an X-band CW-EPR titration experiment was performed. The protein was pre-treated with EDTA (10× the protein concentration) to remove any copper and then buffer-exchanged to remove any EDTA. This copper-free protein sample was tested and, as expected, showed no copper-based signal. Addition of 0.2 equivalents of copper (compared to the protein concentration) showed a single signal in the parallel region, assigned to the copper (II) ion within the histidine brace active site (species 1). Further additions of copper increased this histidine brace copper signal, with concomitant growth of the signal for species 2, already evident after 0.4 equivalents of copper (Additional file 3: Figure S3). These titration experiments were performed at a fixed pH and show that the two species in the EPR spectrum of copper-saturated *Tt*AA10A represent two different Cu-binding sites with slightly different copper-binding affinities, where species 1 is the higher affinity site. Furthermore, the sample of *Tt*AA10A with 0.4 equivalents of Cu was left at 4 °C for 48 h, and its EPR spectrum re-examined. This sample showed no difference in the ratio of copper species, demonstrating that the different binding sites did not arise because of large differences in the kinetics of copper binding.

**Fig. 4:**
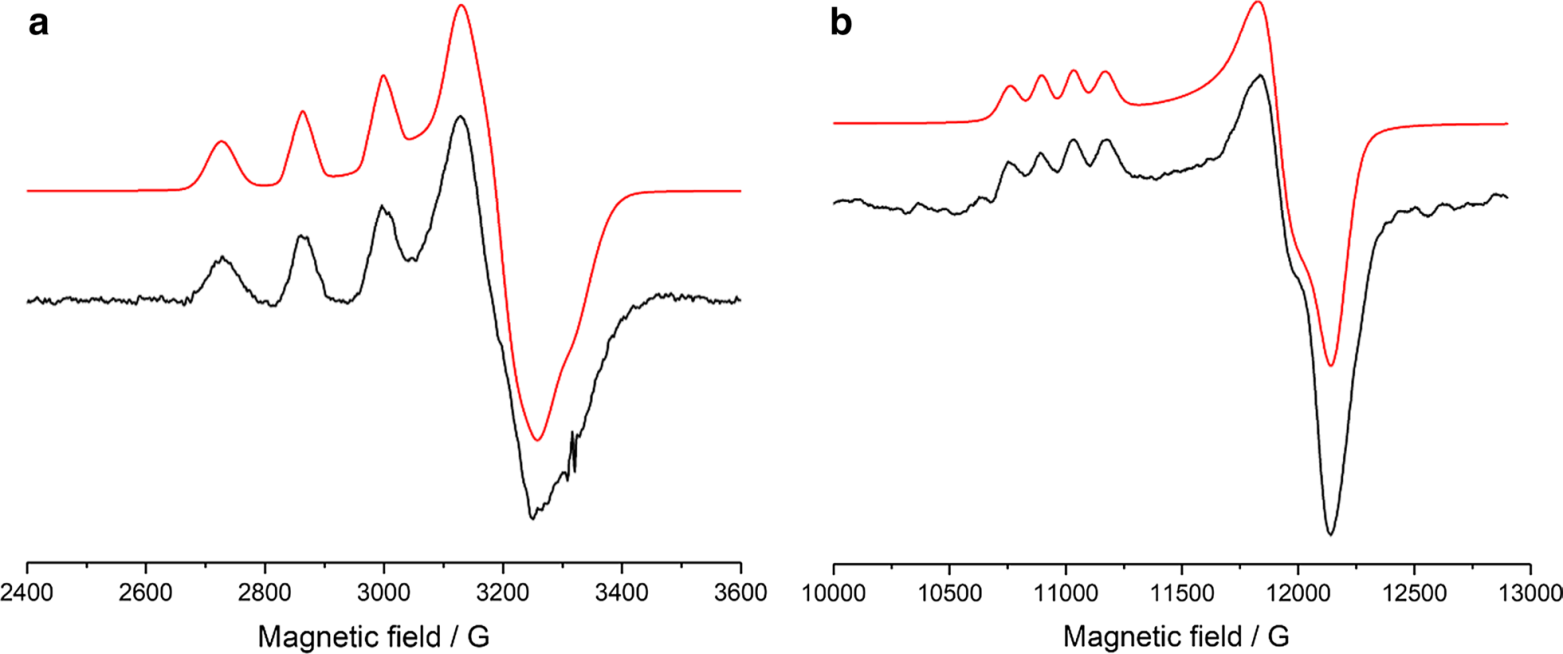
Frozen solution X-band (**a**) and Q-band (**b**) CW-EPR spectra of *Tt*AA10A (species 1). Experimental data in black, simulations in red

Noticeably, in several preparations that we produced, a sample of *Tt*AA10A was isolated which exhibited only a single copper signal in the EPR spectrum. The reasons for this difference in copper stoichiometry of the isolated protein are not clear as these samples were ostensibly prepared using identical conditions as those that afforded *Tt*AA10A with two distinct Cu signals in the X-band EPR spectrum (species 1 and species 2). We were not able to detect any differences in activity for these singly copper occupied preparations relative to previous samples, but we could take advantage of these samples to measure both X-band and Q-band CW-EPR spectra for the active-centre Cu of *Tt*AA10A (Fig. 4) with only the histidine brace occupied, as judged by reference to previous spectra. This sample, therefore, allowed us to perform a simultaneous fit of both the X-band and Q-band spectra to yield more accurate spin Hamiltonian parameters for the copper ion in the histidine brace active site (species 1). These values are reported in Table 2. Addition of PASC to *Tt*AA10A did not cause any change of the EPR spectra (data not shown).

**Table 2:** EPR spin Hamiltonian parameters from simulations of CW X-band and CW Q-band spectra for *Tt*AA10A (species 1) in PBS buffer pH 7.4

	X-band	Q-band
*g* values		
*g*_*x*_	2.048	2.047
*g*_*y*_	2.092	2.088
*g*_*z*_	2.267	2.267
*A*_Cu_ (MHz)		
|*A*_*x*_|	100	100
|*A*_*y*_|	125	125
|*A*_*z*_|	420	425
SHF *A*_N_ principal values (MHz)	38, 40	38, 40
*A*_cu_ strains (MHz)	154, 210, 70	120, 150, 100
Line widths (mT)	0.6, 0.6	4.5, 4.5
Frequency (GHz)	9.3028	34.79

### 3D structure of *Tt*AA10A

**Fig. 5:**
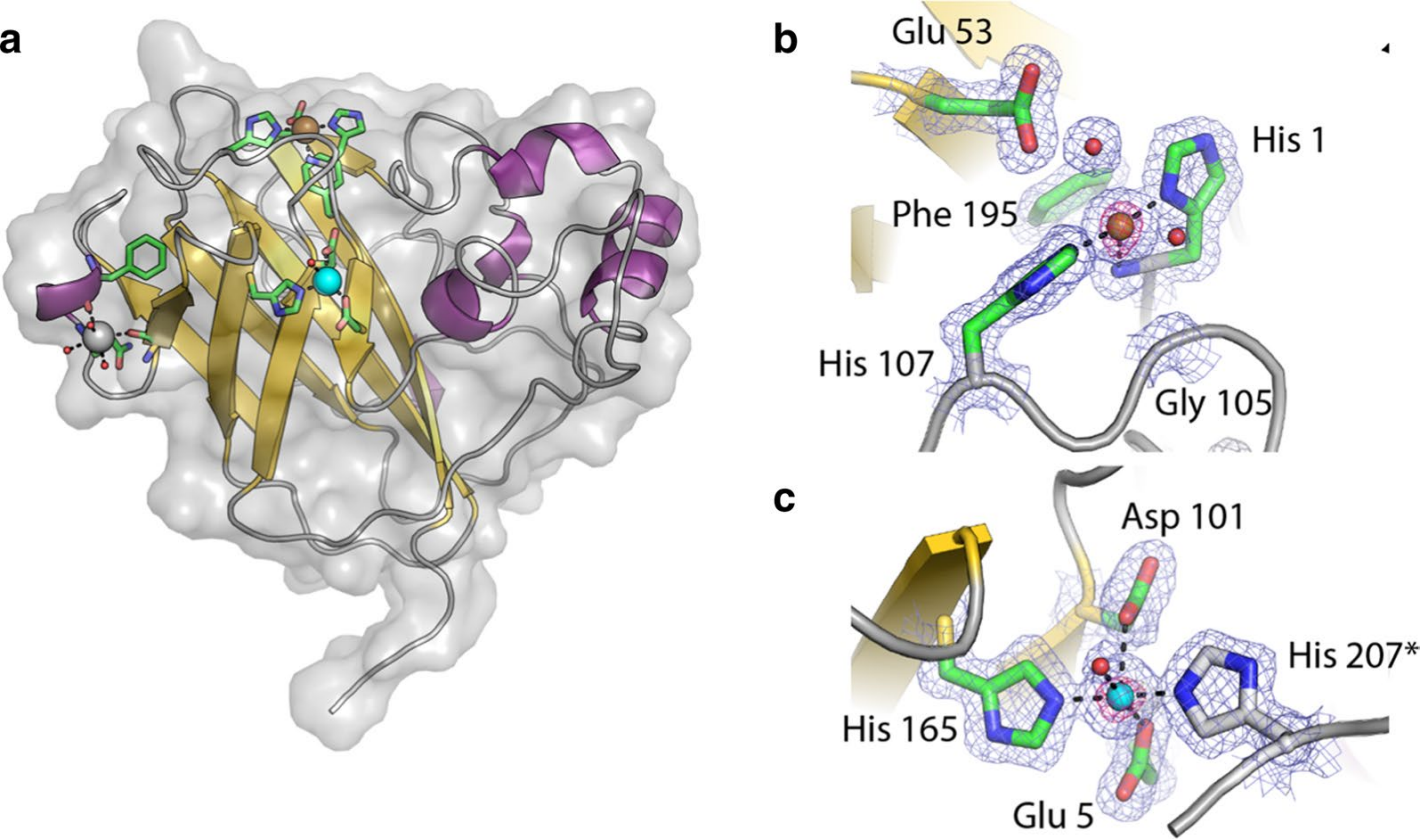
Structural analysis of *Tt*AA10A. **a** The overall structure of *Tt*AA10A is shown as a cartoon coloured by secondary structure with its surrounding surface shown in grey. The histidine brace active site copper is shown as an orange sphere with its coordinating residues displayed as sticks coloured by atom type. The secondary copper site and a separate sodium ion binding site are shown with cyan and grey spheres, respectively, with coordinating residues coloured as for the histidine brace. **b** Close up view of the histidine brace in the enzyme active site. The 2F_obs_–F_calc_ map for the final structure is shown contoured at 1σ as a blue mesh. **c** Close up view of the second copper binding site with the copper ion shown as a cyan sphere. The Strep-Tag-derived histidine which reaches over from a symmetry related molecule to interact with the copper ion is shown with white carbon atoms and is marked with an *. In both **b** and **c**, the difference anomalous map is shown contoured at 4σ as a pink mesh confirming the positions of the copper ions

To gain further insights into the molecular basis for the biochemical properties of *Tt*AA10A, and to probe this, potentially unusual, dual Cu structure, we determined the crystal structure for the recombinantly expressed protein to 1.4 Å resolution (Additional file 4: Table S1). The overall structure revealed a core immunoglobulin-like fold decorated by loops and a helical bundle as typically observed for enzymes from this family (Fig. 5). Indeed, structural comparisons using the DALI server [23] reveal closest structural matches to *Cellvibrio japonicus* AA10A (PDB ID 5fjq) [24] and *Serratia marcescens* CBP21 (PDB ID 2bem) [25] with RMSDs of 2.4 Å and 2.3 Å over 180 and 170 Cα-positions, respectively, representing only 30% identity at the sequence level. Given the high activity of *Tt*AA10A on cellulose, it may be somewhat surprising that the two closest structural matches to this enzyme are chitin-active AA10s. The third closest structural match, however, was the AA10 from *Streptomyces coelicolor* (*Sc*AA10, PDB ID 4oy7 [26]) which is a cellulose-specific AA10 giving an RMSD of 2.5 Å over 160 Cα atoms. *Tt*AA10A and *Sc*AA10 share only 26% sequence identity even though they are active on the same substrate, which further highlights the difficulty in relating LPMO substrate specificity based on sequence and overall structure alone (further discussed in the context of AA9 in Ref. [27]).

As for all LPMOs studied so far (as defined by their activity), the active site of *Tt*AA10A is formed by the “histidine brace” [2] motif which is located at the centre of an almost flat surface (Fig. 5a, b). A single copper ion was modelled at this position coordinated in a typical T-shaped geometry by the amino terminus and side chain of His 1 and the side chain imidazole of His 107. In the axial positions around the active site copper ion, *Tt*AA10A has Phe 195 and Gly 105. These positions are often occupied by a phenylalanine/tyrosine and an alanine side chain, respectively, in other AA10s. The latter has been implicated in creating a steric environment which drives the formation of the distorted active site coordination geometry observed in chitin-specific AA10s in their Cu(II) oxidation state [21, 26, 28–30]. Here, the replacement of Ala by Gly allows the active site copper to adopt a slightly more axial coordination geometry, closer to the one typical of AA9s and cellulose-active AA10s and consistent with the spin Hamiltonian parameters of species 1 in our EPR analysis described earlier, Fig. 4. LPMO crystal structures are often dogged by photo-reduction as a result of radiation damage such that the resting geometry of the active site cannot be directly observed in crystal structures (examples include [21, 28, 30]). Analysis of the sphere surrounding the copper ion in *Tt*AA10A reveals only weak density for a water molecule 2.6 Å from the copper and stronger density for a second water molecule 3.2 Å away hydrogen bonding to Glu 53. These water molecules are too distant from the copper to be considered as directly coordinating. It, therefore, appears that the copper in this enzyme has also undergone photo-reduction to the Cu(I) oxidation state.

Our structure reveals the position of the second copper-binding site that was observed in the EPR spectra. This site is 14.4 Å (Cu…Cu) from the histidine brace copper ion in a large negatively charged patch on the protein surface (Fig. 5a, b; Additional file 5: Figure S4). This second copper ion is directly coordinated by His 165, Glu 5, Asp 101, a water molecule and His 207* which is provided by the Strep-tag from an adjacent molecule in the crystal. Due to the observation of this interaction, we checked the oligomeric state of the protein in solution using SEC–MALLS (Additional file 6: Figure S5). This confirmed that the protein is monomeric, suggesting that the copper–His207* interaction is a crystal artefact (although one that may hint at potential protein–protein interaction). Nonetheless, added to the EPR data, the structure suggests that this second site is occupied in solution, with His 207* likely replaced by a water molecule. Multiple sequence alignment of the top 500 *Tt*AA10A orthologues identified through BlastP searches shows that, while an acidic residue in position 5 is not uncommon among AA10s, residues 101 and 165 are largely conserved only within LPMOs from bacteria that are closely related to *Teredinibacter*.

There has been considerable debate about possible positions in which electron donors, both small molecule and proteinaceous, may bind to LPMOs to allow catalysis when the enzyme is bound to the solid substrate surface (see, for example [19, 31–33]). Indeed, examination of the *Tt*AA10A structure for potential charge transfer pathways using the programme EHPath shows that a clear and rapid hole-hopping pathway with a mean hole residence time of only 20 ms exists between histidine 1 and tyrosine 3 (10 Å separation). Tyrosine 3 is adjacent (5.3 Å) to the second Cu site, thus providing an efficient charge transfer pathway between the two copper sites [34]. Therefore, given the potential charge transfer pathway between the two copper sites, we investigated whether the second metal site (in our case occupied by copper, although we could not displace the Cu with Fe, Ni, Zn and Mn salts) represents a binding site for a proteinaceous redox partner (the binding of another protein to this site is hinted at by the Strep-tag association with a neighbouring molecule in the crystalline lattice), and we attempted to pull down proteins from the *T. turnerae* predicted secretome that may stably interact with *Tt*AA10A using affinity column (StrepTrap HP) immobilised *Tt*AA10A. These experiments (data not shown) did not lead to the isolation of any candidate protein-based activators for *Tt*AA10A, but it cannot be ruled out that an activating enzyme could bind transiently in this region to allow electron transfer to the LPMO and hence the initiation of catalysis. It should be noted, however, that during the structure refinement, we also identified a sodium binding site on the protein surface (Additional file 7: Figure S6). Whether these additional binding sites are a result of the increased charge that this protein may have to be stable in the saline environment in which *T. turnerae* resides remains an open question. Nonetheless, these surface features of *Tt*AA10A may be of interest to enzyme engineers if LPMOs are to be stabilised or adapted to specific conditions to be deployed in industrial bioreactors.

## Discussion

The structural and biochemical characterisation of *Tt*AA10A illuminates the key role of symbiont LPMOs in wood digestion by shipworms. Interestingly, the transcriptome of *L. pedicellatus* contains putative members of two LPMO families (AA10 and AA15, see “Methods” for more details). Predicted AA10 sequences have best matches in the genomes of marine bacteria and are expressed only in the shipworm gills (as reported in previous literature for this and other shipworm species [13, 14]), while putative AA15s have high similarity to sequences from invertebrate genomes (particularly from molluscs) and feature much lower gene expression levels. Shotgun proteomics analysis of the content of the shipworm digestive system has also shown the presence of mature bacterial AA10 LPMOs but no detectable amounts of endogenous AA15s [13]. On the other hand, AA15 LPMOs have high gene expression and protein abundance in the digestive system of the primitive insect *Thermobia domestica*, and were shown to play a role in its ability to digest plant biomass without microbial assistance, while no bacterial AA10s were detected [20]. Unlike *T. domestica*, shipworms appear to have co-opted bacterial AA10 towards cellulose digestion, while the role of endogenous AA15s remains to be determined. Interestingly, the study on *T. domestica* showed that AA15 genes are widespread among invertebrates, including those that do not feed on plant biomass. As such, their ancestral role is most likely to remodel endogenous chitin [20], a structural polysaccharide required for the formation of insect exoskeletons and mollusc shells.

## Conclusions

The in vitro characterisation of *Tt*AA10A revealed the high specificity of this enzyme towards cellulose, a result that we expected based on the presence of a putative cellulose binding domain (CBM10) at the C-terminus of the protein sequence in *T. turnerae*. The products generated by *Tt*AA10A, its EPR spectrum and its X-ray structure, however, were unexpected. While most peaks detected through MALDI-TOF MS are consistent with a mixed C1–C4 oxidation on the cellulose backbone, we also detected a product that could be compatible with higher levels of oxidation/prolonged incubation. The significance of this side reaction in *Tt*AA10A is beyond the scope of the current manuscript. However, both X-ray structural features and EPR spectra support the existence of a second copper-binding site (distinct from the conserved histidine brace) that could potentially influence LPMO mechanism of action, electron donation, and thereby the nature of the generated products. The identification of a sodium-binding site on the surface of *Tt*AA10A is also of interest, as it might play a role in stabilising the enzyme and help it cope with the marine environment conditions, thus opening up new potential opportunities in industrial applications requiring high salt. Shipworm symbionts, therefore, offer an exciting environmental niche in which to hunt for LPMOs with unusual properties.

## Methods

### Heterologous gene expression and protein purification

The LPMO domain of *TtAA10A* (without its CBM) was codon optimised and cloned using the In-Fusion HD cloning kit (Takara, Saint-Germain-en-Laye, France) into a modified pET22b vector containing at the N-terminus the pelB leader sequence to direct protein production to the periplasm, and a C-terminal Strep-tag. The construct was transformed into Tig Chaperone *E. coli* cells (Pgro7 Chaperone set Takara). LB liquid medium containing ampicillin (100 μg/mL) and chloramphenicol (35 μg/mL) was inoculated with starter culture, and l-arabinose (0.5 g/L final concentration) was added to induce expression of the Tig chaperone. Cultures were grown at 37 °C until the OD was approximately 0.6. The cultures were left to cool slightly before IPTG was added to a final concentration of 1 mM, and they were incubated overnight at 16 °C with shaking.

Cultures were harvested by centrifugation, 5000*g* for 30 min. For each 100 mL of original culture the pellet was gently resuspended in 5 mL of ice cold 50 mM Tris HCl buffer pH 8 with 20% *v/v* sucrose and left on ice for 30 min with occasional mixing. The cell suspension was centrifuged again at 8000 rpm for 10 min, and the cells subjected to osmotic shock; pellet was resuspended in ice-cold 1 mM MgSO_4_ (5 mL per 100 mL of initial culture) plus AEBSF protease inhibitor, and left on ice for 30 min with occasional mixing. The suspension was centrifuged again, and the supernatant collected, filtered and diluted to make up a 1× PBS solution (using stock 10× PBS, pH 7.4).

The sample was loaded onto a strep column (GE Healthcare) pre-equilibrated in 1× PBS pH 7.4. The column was washed with 1× PBS buffer for 5 column volumes, followed by protein elution using 5 column volumes of 1× PBS pH 7.4, 2.5 mM desthiobiotin.

The dilute *Tt*AA10A-strep protein sample was copper loaded by incubation with excess CuSO_4_ (1 mM final concentration equivalent to 10× the protein concentration) at 4 °C overnight. To remove excess desthiobiotin and unbound copper, the protein sample was filtered, concentrated and passed through a HiLoadTM 16/60 Superdex 75 gel filtration column (Ge Healthcare) equilibrated with PBS pH 7.4.

### In vitro activity assays

Activity assays were carried out on microcrystalline cellulose (Avicel), squid pen chitin, shrimp chitin, glucomannan (low viscosity from konjac, Megazyme), pachyman (Megazyme), xyloglucan (from tamarind, Megazyme), lichenan (from icelandic moss, Megazyme), galactan (from lupin, Megazyme), galactomannan (from carob, Megazyme), mannan (borohydride reduced, Megazyme), cellohexaose (Megazyme, used in final concentration of 40 µM), corn starch (Sigma) and beechwood xylan (Serva).

Typical reactions for LPMO characterization through mass spectrometry were carried out by mixing 4 mg/mL substrate with purified *Tt*AA10A (2 μM) and 4 mM gallic acid in a total volume of 100 μL in 2 mL plastic reaction tubes for 24 h. All reactions analysed via MALDI-TOF MS were carried out in 50 mM ammonium acetate buffer pH 6 and incubated at 28 °C shaking at 600 rpm and the supernatant used for analysis. Samples were analysed by MALDI-TOF MS as described in [20].

Reactions used for product quantification and boosting experiments with *Tt*AA10A were typically carried out in 50 mM sodium phosphate buffer pH 6 in triplicates of 100 μL each for 3 h at 600 rpm at 28 °C. Each reaction contained 2 μM purified LPMO, 4 mg/mL substrate, and 1 mM gallic acid. Commercial GH6 (cat. number E-CBHIIM, Megazyme, 0.8 mU) and GH9 (cat. number CZ03921, NZYTech, 10 µg) were added to 100 μL reactions. After 3-h incubation, 400 μL of ethanol was added to stop the reaction, spun down and 400 μL of supernatant was transferred to new plastic tubes, dried down and re-suspended in 80 μL of pure water, filtered and analysed via HPAEC as previously described [20].

### Electron paramagnetic resonance (EPR) spectroscopy

Frozen solution CW X- and Q-band EPR spectra of *Tt*AA10A were collected at 165 K on a Bruker micro EMX spectrometer operating at ~ 9.3 GHz, modulation amplitude of 4 G and 10.02 mW microwave power or at 77 K on a Jeol JES-X320 spectrometer operating at ~ 34.7 GHz with microwave power of 1 mW, respectively. Protein samples used during single EPR analysis were all in the concentration range 100–200 μM for X-band and 1 mM for Q-band, in 1X PBS buffer at pH 7.4. Simulations of the collected spectra were carried out in Easy Spin 5.2.6 [35] integrated into MatLab 2016a software to determine the *g* and A-tensor parameters. Accurate determination of the spin Hamiltonian parameters for species 1 was obtained by simultaneous fitting of both X- and Q-band spectra. The superhyperfine coupling values for the nitrogen atoms could not be determined accurately, although it was noted that the fitting was highly improved by addition of two nitrogen atoms with coupling in the range 35–40 MHz. Accurate determination of the *g*_*x*_, *g*_*y*_, |*A*_*x*_| and |*A*_*y*_| values for species 2 was not possible due to the overlap of the two species in the perpendicular region. EPR of different batches of protein showed that some samples contained two copper species, whilst other samples contained only one. EPR Copper titrations (CuSO_4_ 1 M) were carried out on protein free of copper (10 mM EDTA treated, followed by extensive buffer exchange), with spectra taken before the titration was started confirming the lack of coordinated copper in the protein. Copper was added to the protein solution whilst contained within the EPR tubes, in additions of 0.2 equivalents (to the concentration of the protein in the sample). Spectra were measured after each addition of copper solution. Raw EPR data is available on request through Research Data York with DOI: 10.15124/4d239806-7529-45fc-95a1-c14bcadc24d2.

### *Tt*AA10A crystallisation, structure solution and refinement

*Tt*AA10A was screened for crystallisation at 7 mg/mL using the Hampton HT (Hampton Research) and PEG/Ion (Qiagen) screens in 96 well sitting drops using Mosquito robotics (TTP Labtech). Initial hits were obtained in the PEG/Ion screen condition B8 (0.2 M magnesium formate dehydrate and 20% *w/v* PEG 3350, pH 7), which were subsequently optimised in hanging drops, screening across a narrow range of concentrations for both magnesium formate dehydrate and PEG 3350. A single crystal was cryo-cooled for data collection by plunging in liquid nitrogen without the addition of cryo-protectant.

X-ray diffraction data were collected at Diamond Light Source, beamline I04 and were processed in the CCP4i2 pipeline [36] to 1.4 Å resolution (see Additional file 2: Table S1, for processing statistics). The structure was determined by single wavelength anomalous dispersion (SAD) in CRANK2 [37] using the anomalous signal from protein-bound copper ions. Briefly, SHELX [38] was used to solve the phase problem and to generate an initial structural model which was further elaborated upon in BUCCANEER [39]. The final model was generated using iterative cycles of rebuilding and refinement in COOT [40] and REFMAC [41], respectively (Additional file 2: Table S1). The final model and accompanying structure factors have been deposited in the Protein Data Bank with accession code 6RW7.

### Data mining

The online tool DBCAN2 [42] was used to analyse published transcriptomic data from the shipworm *Lyrodus pedicellatus* [13] and identify putative AA10 and AA15 sequences.

## Supplementary information

**Additional file 1: Figure S1.** MALDI-TOF MS analysis of in vitro negative control activity assays with purified *Tt*AA10A, under the same experimental conditions as in Fig. 2a. The panels show spectra of products obtained after incubation of 4 mg/mL Avicel (**a**), Avicel with 4 mM gallic acid (**b**) and Avicel with 2 µM *Tt*AA10A (**c**). The spectra show no detectable amounts of native or oxidised cello-oligosaccharides. Relative intensity represents 1.23 × 10^3^.

**Additional file 2: Figure S2.** HPAEC chromatograms showing the release of cellobiose from Avicel during boosting experiments with *Tt*AA10A, commercial GH6 and gallic acid. The identity and concentration of cellobiose (retention time approx. 4.5 mins) was determined by analysis of a commercial standard (Std). The small amount of additional cellobiose released with LPMO in the absence of external reducing substrate is assumed to be derived from low quantities of unknown reductant, as has been observed on other systems such as AA13 [43] and AA15 [20].

**Additional file 3: Figure S3.** EPR Cu titration reveals the presence of two distinct Cu sites. EPR copper titration experiment in which the protein was pre-treated with EDTA to remove the copper (as shown by the lack of EPR signal). Addition of 0.2 equivalents of Cu (to the concentration of protein) produced a single Cu species indicative of the histidine brace. Addition of a further 0.2 equivalents caused a change in the spectra with appearance of a second set of hyperfine peaks in the parallel region. The clearest change is seen after 0.6 equivalents of Cu have been added, where multiple peaks in the spectra indicate two Cu species. Loss of resolution in the last two spectra may be due to signal dilution.

**Additional file 4: Table S1.** *Tt*AA10A Data collection and refinement statistics.

**Additional file 5: Figure S4.** Electrostatic Surface Potential for *Tt*AA10A. The electrostatic charge distribution for *Tt*AA10A has been mapped onto the protein surface using the APBS plug-in for PyMol at ± 10 KBT/e. The histidine brace, secondary copper binding site and sodium binding site are outlined with black lines showing the negatively charged areas to which the ions bind.

**Additional file 6: Figure S5.** SEC-MALLS analysis of *Tt*AA10A, where the solid red line indicates the refractive index, the dashed line is the light scattering (these are essentially identical so indistinguishable), dotted line the UV response at 280 nm. The central line inside the peak is representative of the molar mass (2.44 × 10^4^ Da), indicating that *Tt*AA10A forms a monomer in solution.

**Additional file 7: Figure S6.** The Na^+^ Site on the *Tt*AA10A Surface. The modelled sodium ion is shown as a grey sphere with the groups that coordinate it shown as sticks coloured by atom type. The 2*F*_obs_–*F*_calc_ map is shown as a blue wire mesh contoured at 1σ. The sodium ion is octahedrally coordinated with three main chain carbonyl groups and three water molecules. Sodium was assigned at this site based on the coordinating bond lengths and this was the ion that gave a B-factor following refinement closest to the surrounding protein atoms.

## Data Availability

Coordinates and observed data have been deposited in the PDB with accession code 6RW7.

## Abbreviations

AA: auxiliary activity
CBM: carbohydrate-binding module
DP: degree of polymerisation
EPR: electron paramagnetic resonance
GH: glycoside hydrolase
HPAEC: high-pressure anion-exchange chromatography
HPLC: high-pressure liquid chromatography
(L)PMO: (lytic) polysaccharide monooxygenase
MALDI (TOF): matrix-assisted laser desorption/ionisation (time of flight)
PASC: phosphoric acid swollen cellulose
PBS: phosphate-buffered saline
PDB: protein databank
PEG: polyethylene glycol
SAD: single-wavelength anomalous dispersion
