# Supplementary material for: Discovery, activity and characterisation of an AA10 lytic polysaccharide oxygenase from the shipworm symbiont Teredinibacter turnerae
Source: Biotechnol Biofuels. 2019 Sep 30;12:232. doi: 10.1186/s13068-019-1573-x (PMC6767633; doi:10.1186/s13068-019-1573-x)
Supplement: Supplementary file 1 — Additional file 1: Figure S1. MALDI-TOF MS analysis of in vitro negative control activity assays with purified TtAA10A, under the same experimental conditions as in Fig. 2a. The panels show spectra of products obtained after incubation of 4 mg/mL Avicel (a), Avicel with 4 mM gallic acid (b) and Avicel with 2 µM TtAA10A (c). The spectra show no detectable amounts of native or oxidised cello-oligosaccharides. Relative intensity represents 1.23 × 10^3^. [file 13068_2019_1573_MOESM1_ESM.docx]

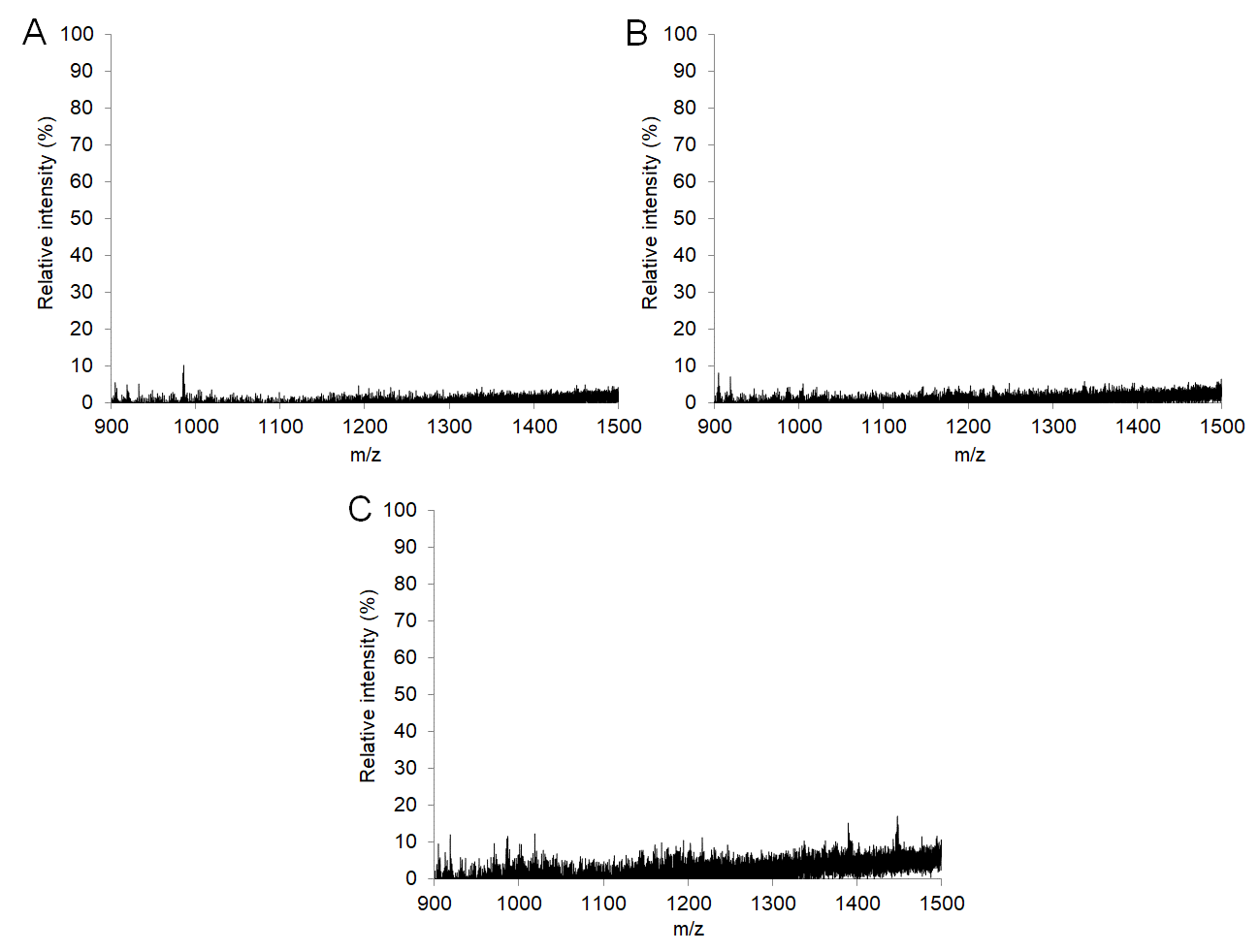


**Additional File 1, Figure S1**. MALDI-TOF MS analysis of in vitro negative control activity assays with purified *Tt*AA10A, under the same experimental conditions as in Figure 2A. The panels show spectra of products obtained after incubation of 4 mg mL^-1^ Avicel (A), Avicel with 4 mM gallic acid (B) and Avicel with 2 µM *Tt*AA10A (C). The spectra show no detectable amounts of native or oxidized cello-oligosaccharides. Relative intensity represents 1.23 x 10^3^.
