# Supplementary material for: Discovery, activity and characterisation of an AA10 lytic polysaccharide oxygenase from the shipworm symbiont Teredinibacter turnerae
Source: Biotechnol Biofuels. 2019 Sep 30;12:232. doi: 10.1186/s13068-019-1573-x (PMC6767633; doi:10.1186/s13068-019-1573-x)
Supplement: Supplementary file 2 — Additional file 2: Figure S2. HPAEC chromatograms showing the release of cellobiose from Avicel during boosting experiments with TtAA10A, commercial GH6 and gallic acid. The identity and concentration of cellobiose (retention time approx. 4.5 mins) was determined by analysis of a commercial standard (Std). The small amount of additional cellobiose released with LPMO in the absence of external reducing substrate is assumed to be derived from low quantities of unknown reductant, as has been observed on other systems such as AA13 [43] and AA15 [20]. [file 13068_2019_1573_MOESM2_ESM.docx]

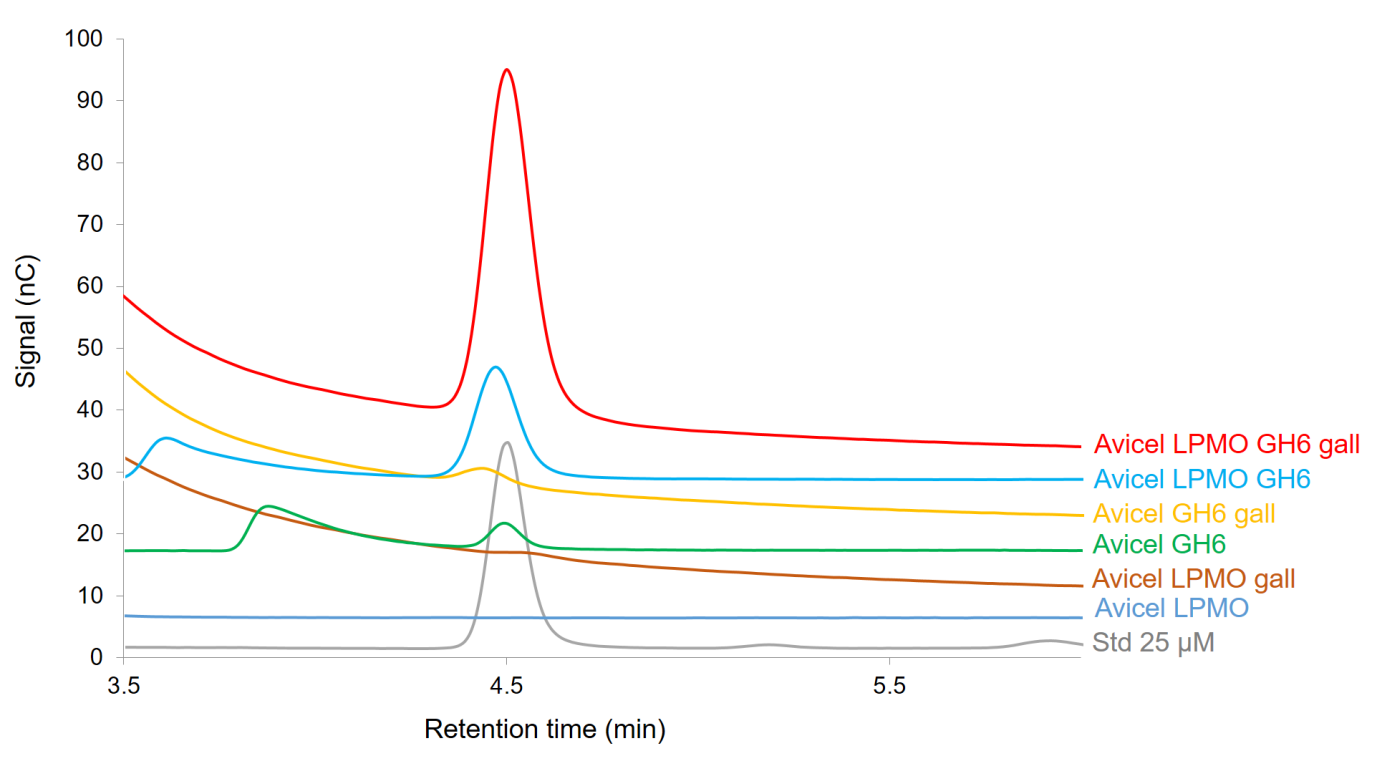


**Additional File 2, Figure S2**. HPAEC chromatograms showing the release of cellobiose from Avicel during boosting experiments with *Tt*AA10A, commercial GH6 and gallic acid. The identity and concentration of cellobiose (retention time approx. 4.5 mins) was determined by analysis of a commercial standard (Std). The small amount of additional cellobiose released with LPMO in the absence of external reducing substrate is assumed to be derived from low quantities of unknown reductant, as has been observed on other systems such as AA13 [43] and AA15 [20].
