# Supplementary material for: Discovery, activity and characterisation of an AA10 lytic polysaccharide oxygenase from the shipworm symbiont Teredinibacter turnerae
Source: Biotechnol Biofuels. 2019 Sep 30;12:232. doi: 10.1186/s13068-019-1573-x (PMC6767633; doi:10.1186/s13068-019-1573-x)
Supplement: Supplementary file 3 — Additional file 3: Figure S3. EPR Cu titration reveals the presence of two distinct Cu sites. EPR copper titration experiment in which the protein was pre-treated with EDTA to remove the copper (as shown by the lack of EPR signal). Addition of 0.2 equivalents of Cu (to the concentration of protein) produced a single Cu species indicative of the histidine brace. Addition of a further 0.2 equivalents caused a change in the spectra with appearance of a second set of hyperfine peaks in the parallel region. The clearest change is seen after 0.6 equivalents of Cu have been added, where multiple peaks in the spectra indicate two Cu species. Loss of resolution in the last two spectra may be due to signal dilution. [file 13068_2019_1573_MOESM3_ESM.docx]

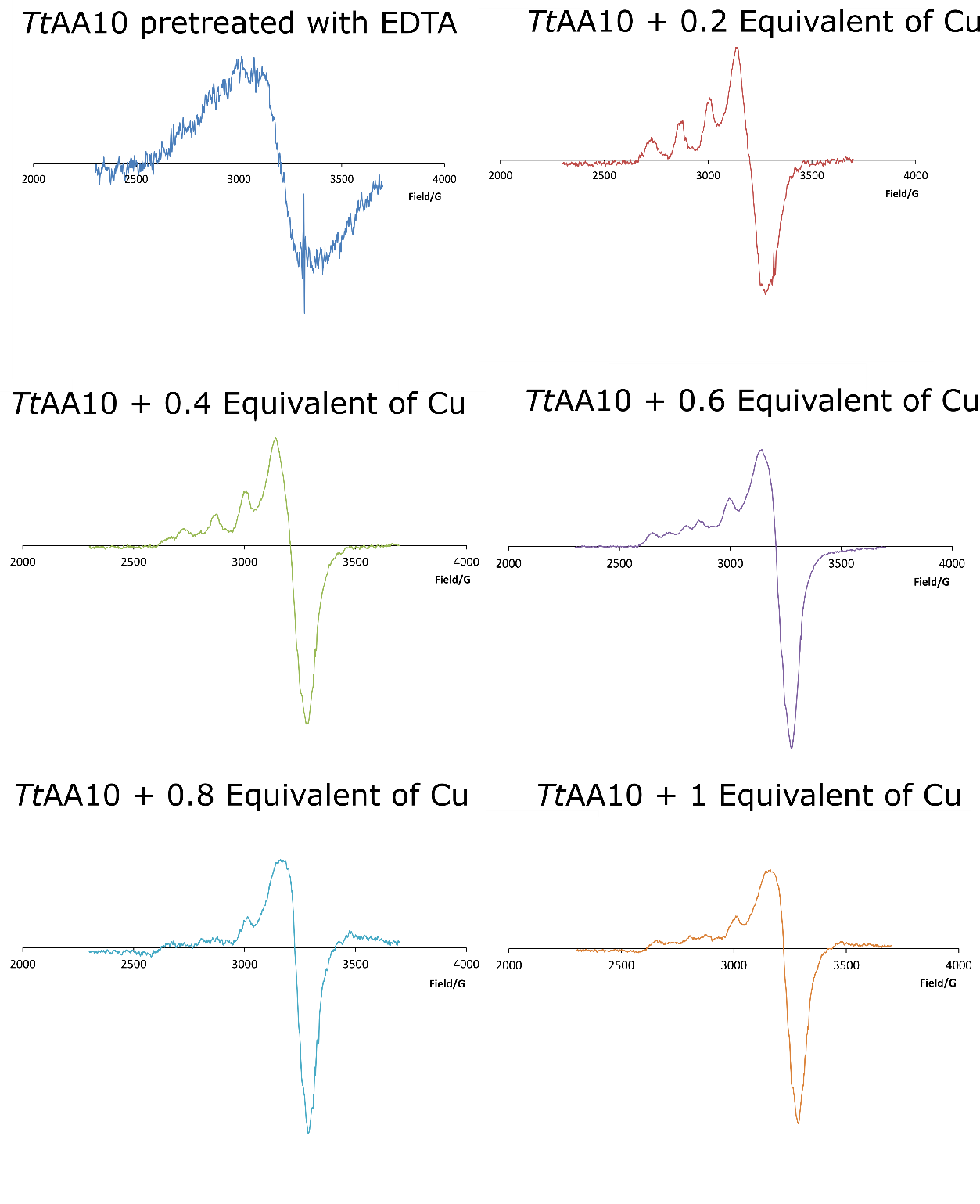


**Additional File 3, Figure S3. EPR Cu titration reveals the presence of two distinct Cu sites.** EPR copper titration experiment in which the protein was pre-treated with EDTA to remove the copper (as soon by the lack of EPR signal). Addition of 0.2 equivalents of Cu (to the concentration of protein) produced a single Cu species indicative of the histidine brace. Addition of a further 0.2 equivalents caused a change in the spectra with appearance of a second set of hyperfine peaks in the parallel region. The clearest change is seen after 0.6 equivalents of Cu have been added, where multiple peaks in the spectra indicate two Cu species. Loss of resolution in the last two spectra may be due to signal dilution.
