# Supplementary material for: Discovery, activity and characterisation of an AA10 lytic polysaccharide oxygenase from the shipworm symbiont Teredinibacter turnerae
Source: Biotechnol Biofuels. 2019 Sep 30;12:232. doi: 10.1186/s13068-019-1573-x (PMC6767633; doi:10.1186/s13068-019-1573-x)
Supplement: Supplementary file 4 — Additional file 4: Table S1. TtAA10A Data collection and refinement statistics. [file 13068_2019_1573_MOESM4_ESM.docx]

**Additional File 4, Table S1. *Tt*AA10A Data collection and refinement statistics**

|  | *Tt*AA10A |
| --- | --- |
| **Data collection** |  |
| Space group | P2_1_ |
| Cell dimensions |  |
| *a*, *b*, *c* (Å) | 36.6, 62.5, 43.2 |
| α, β, γ (°) | 90.0, 99.1, 90.0 |
| Resolution (Å) | 36.18 – 1.40 (1.42 – 1.40) * |
| *R*_merge_ | 0.072 (0.796) |
| *R_pim_* | 0.041 (0.548) |
| *CC(1/2)* | 0.998 (0.606) |
| *I* / σ*I* | 12.3 (1.3) |
| Completeness (%) | 99.5 (95.6) |
| Multiplicity | 3.9 (2.7) |
|  |  |
| **Refinement** |  |
| Resolution (Å) | 31.28 – 1.40 |
| No. reflections | 37716 |
| *R*_work_ / *R*_free_ | 0.146/0.176 |
| *B*-factors (Å^2^) |  |
| Protein | 13.3 |
| Ions | 15.3 |
| Water | 24.5 |
| R.m.s. deviations |  |
| Bond lengths (Å) | 0.025 |
| Bond angles (°) | 2.53 |
| PDB ID | **6RW7** |

*Values in parentheses are for highest-resolution shell.
