# Supplementary material for: Discovery, activity and characterisation of an AA10 lytic polysaccharide oxygenase from the shipworm symbiont Teredinibacter turnerae
Source: Biotechnol Biofuels. 2019 Sep 30;12:232. doi: 10.1186/s13068-019-1573-x (PMC6767633; doi:10.1186/s13068-019-1573-x)
Supplement: Supplementary file 5 — Additional file 5: Figure S4. Electrostatic Surface Potential for TtAA10A. The electrostatic charge distribution for TtAA10A has been mapped onto the protein surface using the APBS plug-in for PyMol at ± 10 KBT/e. The histidine brace, secondary copper binding site and sodium binding site are outlined with black lines showing the negatively charged areas to which the ions bind. [file 13068_2019_1573_MOESM5_ESM.docx]

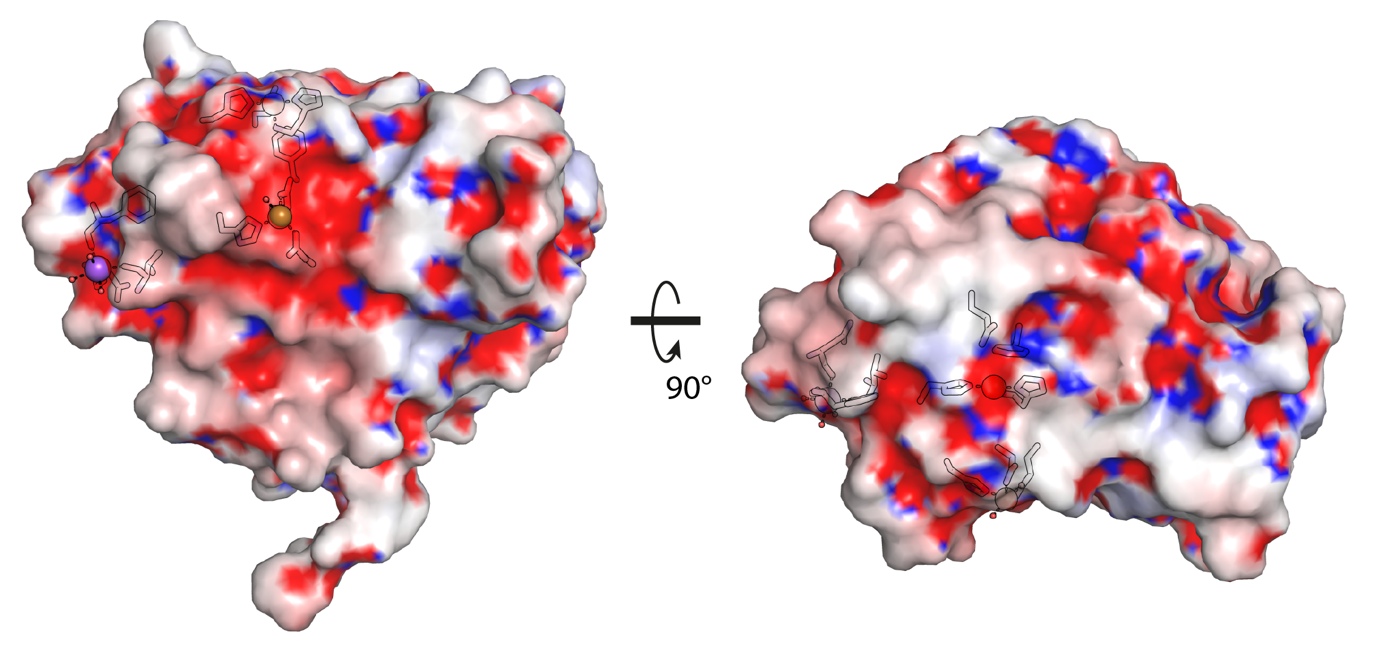


**Additional File 5, Figure S4. Electrostatic Surface Potential for *Tt*AA10A.** The electrostatic charge distribution for *Tt*AA10 has been mapped onto the protein surface using the APBS plug-in for PyMol at ±10 KBT/e. The histidine brace, secondary copper binding site and sodium binding site are outlined with black lines showing the negatively charged areas to which the ions bind.
