# Supplementary material for: Discovery, activity and characterisation of an AA10 lytic polysaccharide oxygenase from the shipworm symbiont Teredinibacter turnerae
Source: Biotechnol Biofuels. 2019 Sep 30;12:232. doi: 10.1186/s13068-019-1573-x (PMC6767633; doi:10.1186/s13068-019-1573-x)
Supplement: Supplementary file 6 — Additional file 6: Figure S5. SEC-MALLS analysis of TtAA10A, where the solid red line indicates the refractive index, the dashed line is the light scattering (these are essentially identical so indistinguishable), dotted line the UV response at 280 nm. The central line inside the peak is representative of the molar mass (2.44 × 10^4^ Da), indicating that TtAA10A forms a monomer in solution. [file 13068_2019_1573_MOESM6_ESM.docx]

**
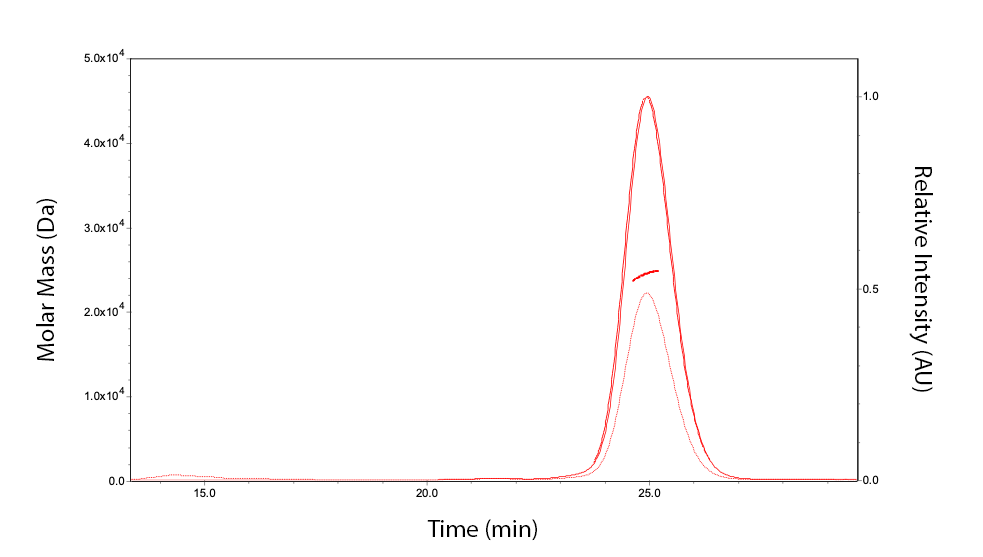
**

**Additional File 6, Figure S5** SEC-MALLS analysis of *Tt*AA10A, where the solid red line indicates the refractive index, the dashed line is the light scattering (these are essentially identical so indistinguishable), dotted line the UV response at 280 nm. The central line inside the peak representative of the molar mass (2.44 x10^4^ Da), indicating that *Tt*AA10A forms a monomer in solution.
