# Supplementary material for: Discovery, activity and characterisation of an AA10 lytic polysaccharide oxygenase from the shipworm symbiont Teredinibacter turnerae
Source: Biotechnol Biofuels. 2019 Sep 30;12:232. doi: 10.1186/s13068-019-1573-x (PMC6767633; doi:10.1186/s13068-019-1573-x)
Supplement: Supplementary file 7 — Additional file 7: Figure S6. The Na+ Site on the TtAA10A Surface. The modelled sodium ion is shown as a grey sphere with the groups that coordinate it shown as sticks coloured by atom type. The 2Fobs–Fcalc map is shown as a blue wire mesh contoured at 1σ. The sodium ion is octahedrally coordinated with three main chain carbonyl groups and three water molecules. Sodium was assigned at this site based on the coordinating bond lengths and this was the ion that gave a B-factor following refinement closest to the surrounding protein atoms. [file 13068_2019_1573_MOESM7_ESM.docx]

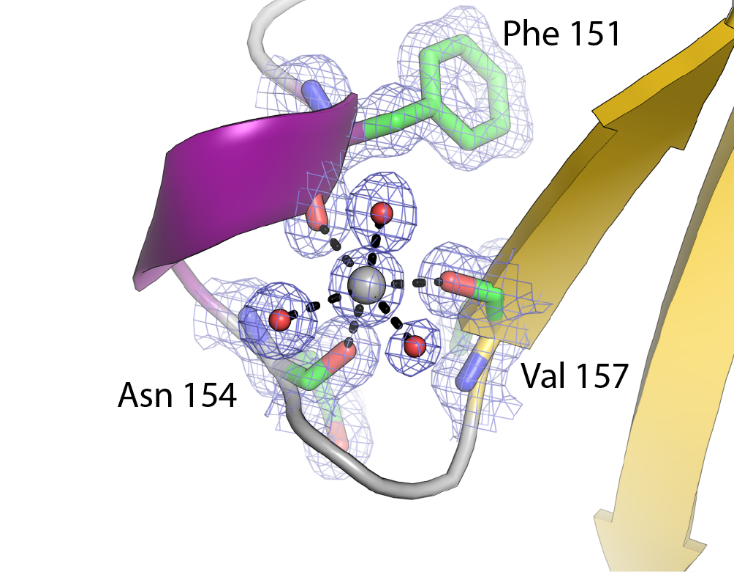


**Additional File 7, Figure S6. The Na^+^ Site on the *Tt*AA10A Surface.** The modelled sodium ion is shown as a grey sphere with the groups that coordinate it shown as sticks coloured by atom type. The 2F_obs_-F_calc_ map is shown as a blue wire mesh contoured at 1σ. The sodium ion is octahedrally coordinated with three main chain carbonyl groups and three water molecules. Sodium was assigned at this site based on the coordinating bond lengths and this was the ion that gave a B-factor following refinement closest to the surrounding protein atoms.
